# Induction of Suppressor Cells and Increased Tumor Growth following Chronic Psychosocial Stress in Male Mice

**DOI:** 10.1371/journal.pone.0159059

**Published:** 2016-07-08

**Authors:** Dominic Schmidt, Daniel Peterlik, Stefan O. Reber, Anja Lechner, Daniela N. Männel

**Affiliations:** 1 Institute of Immunology, University of Regensburg, Regensburg, Germany; 2 Institute of Zoology, Laboratory of Molecular and Cellular Neurobiology, University of Regensburg, Regensburg, Germany; 3 Institute of Zoology, Department of Behavioral and Molecular Neurobiology, University of Regensburg, Regensburg, Germany; National Institute of Allergy and Infectious Diseases, UNITED STATES

## Abstract

To study the impact of psychosocial stress on the immune system, male mice were subjected to chronic subordinate colony housing (CSC), a preclinically validated mouse model for chronic psychosocial stress. CSC substantially affected the cell composition of the bone marrow, blood, and spleen by inducing myelopoiesis and enhancing the frequency of regulatory T cells in the CD4 population. Expansion of the myeloid cell compartment was due to cells identified as immature inflammatory myeloid cells having the phenotype of myeloid-derived suppressor cells of either the granulocytic or the monocytic type. Catecholaminergic as well as TNF signaling were implicated in these CSC-induced cellular shifts. Although the frequency of regulatory cells was enhanced following CSC, the high capacity for inflammatory cytokine secretion of total splenocytes indicated an inflammatory immune status in CSC mice. Furthermore, CSC enhanced the suppressive activity of bone marrow-derived myeloid-derived suppressor cells towards proliferating T cells. In line with the occurrence of suppressor cell types such as regulatory T cells and myeloid-derived suppressor cells, transplanted syngeneic fibrosarcoma cells grew better in CSC mice than in controls, a process accompanied by pronounced angiogenesis and clustering of immature myeloid cells in the tumor tissue. In addition, tumor implantation after CSC reinforced the CSC-induced increase in myeloid-derived suppressor cells and regulatory T cell frequencies while the CSC-induced cellular changes eased off in mice without tumor. Together, our data suggest a role for suppressor cells such as regulatory T cells and myeloid-derived suppressor cells in the enhanced tumor growth after chronic psychosocial stress.

## Introduction

The two major stress systems of an organism, namely the hypothalamus-pituitary-adrenal (HPA) axis and the sympathetic nervous system (SNS), interact with the immune system in a complex manner. While acute stress enhances immune responses, studies employing repeated or chronic stressors often demonstrate a pronounced and long lasting suppressive effect on immune function, paralleled among others by an increased susceptibility to infections (reviewed in [1]). This is in line with the well-known anti-inflammatory effects of glucocorticoids and the fact that chronic stress has been linked to hypercorticism [2]. However, accumulating evidence from human and animal studies suggests chronic stressors, if severe enough, to promote decreased rather than increased glucocorticoid signaling caused by hypocorticism and/or glucocorticoid resistance [3;4]. In accordance with this lack of adequate immune regulation, chronic stress has also been linked to increased transcription of inflammatory genes and myelopoiesis [5] and a long-lasting (up to two weeks) enhancement of pro-inflammatory and suppression of anti-inflammatory cytokine production [6]. Although seeming contradictory at first glance, given these immune-enhancing effects, chronic stress is an accepted risk factor for cancer [7;8]. Accumulating data from animal studies further support a prominent role for the SNS in chronic stress-induced myelopoiesis and migration of myeloid cells into the periphery [9;10], as well as in tumor progression (reviewed in [11]).

Chronic subordinate colony housing (CSC) is an established model for chronic psychosocial stress in male mice, in which subordinate CSC mice are housed together with a larger dominant male for 19 consecutive days [12]. In contrast to single-housed control (SHC) mice, CSC mice are more anxious, show increased plasma norepinephrine levels (i.e. increased activity of the SNS), develop spontaneous colitis, and a reduction in glucocorticoid signaling mediated by both hypocorticism and glucocorticoid resistance [12;13]. In addition, CSC mice have a higher risk of developing colorectal cancer [14] and are sensitized towards inflammatory challenges as shown by the aggravation of a dextran sodium sulfate (DSS)-induced colitis [15]. Analysis of peripheral immune responses after CSC revealed a generalized activation of all T cell subsets with the T helper (Th) cells shifting towards higher production capacity for Th1, Th2, and Th17 cytokines [13]. These findings support the idea that chronic psychosocial stress induced by CSC promotes both immune activation and carcinogenesis.

During an ongoing immune response, regulatory immune cells such as regulatory T (Treg) cells and myeloid-derived suppressor cells (MDSC) are generated in order to resolve the inflammation and avoid tissue damage [16;17]. Treg cells represent a subpopulation of CD4^+^ T cells and are identified by their expression of the transcription factor Foxp3 [18;19]. The mechanisms by which Treg cells suppress effector functions of T cells has been reviewed in detail elsewhere [20]. On the other hand, myeloid cells represent a heterogeneous population of effector cells, belong to the innate immune system, are generated in the bone marrow, and—given their prominent role in eradication of pathogens via phagocytosis and antigen presentation—constitute a first line of defense during infections. In addition, they are also important immune-regulators [21;22]. Immature myeloid cells suppressing T cell proliferation were first detected in tumor patients and tumor-bearing mice and named MDSC [23;24]. The suppressive mechanisms of MDSC include generation of nitric oxide (NO), reactive oxygen species, depletion of arginine and down-regulation of the T cell receptor complex ζ chain (reviewed in [25]). In addition, MDSC support expansion of Treg cells by release of IL-10 [26]. Mature granulocytes and immature myeloid cells are characterized by the cell surface markers CD11b and Gr1, whereby the latter are less granular and either of mononuclear or granulocyte-like shape depending on the degree of their Gr1 expression level [27]. The immature myeloid cell populations found in cancer tissue were therefore further differentiated into polymorphonuclear cells expressing Ly6G^high^ Ly6C^low^ (PMN-MDSC) [28] and Ly6G^neg^Ly6C^high^ highly suppressive monocyte-like cells (MO-MDSC) [29]. Meanwhile it has been shown that both types of immature myeloid cells are also induced during bacterial infections [30;31]. Thus, MDSC seem to provide a cellular link between immune activation and cancer [32].

The aim of the present study was to investigate the link between chronic psychosocial stress-induced immune activation on the one and tumor progression on the other hand. The effects of CSC on cells of the lymphoid and myeloid cell compartment with focus on number and functionality of MDSC were assessed. CSC induced cell shifts typical for acute inflammation such as myelopoiesis with the appearance of MDSC. In addition, as in ongoing inflammatory reactions, the frequency of Treg was transiently increased. Enhanced growth of transplanted syngeneic fibrosarcoma tumor cells was seen in CSC mice possibly referring to the suppressed immune status of the stressed mice.

## Material and Methods

### Animals

Male C57BL/6N mice weighing 19–22 g were purchased from Charles River (Sulzfeld, Germany), TNF-deficient mice (B6.TNF^−/−^) [33] and TNFR2-deficient mice (C57BL/6-*Tnfrsf1b*^*tm1Mwm*^) [34] were bred in the animal facility of the University of Regensburg. Deficiency of TNF, TNFR1 and TNFR2 expression was verified by PCR. All mice were housed under standard laboratory conditions in the animal facility of the University of Regensburg. The male offspring of CD1 female mice (bred at the Max Planck Institute of Psychiatry in Munich (R. Landgraf) for high anxiety-related behavior) and male C57BL/6 mice or CD1 mice were used as dominant animals (body weight: 30-35g). Those were kindly provided by I.D. Neumann (University of Regensburg) or commercially obtained (Charles River). Mice were killed for *post mortem* analysis by cervical dislocation. All research involving animals have the approval from the authors' Institutional Animal Care and Use Committee (IACUC, Regierung von Unterfranken, Würzburg, Germany, permit number AZ 54–2532.1-17/14), and have been conducted according to applicable national and international guidelines.

### Chronic subordinate colony housing

Mice were individually housed for one week prior to starting CSC [12]. Four experimental animals were housed together with a larger dominant male (30–35 g) in a polycarbonate observation cage (38 x 22 x35 cm) for 19 consecutive days. Dominant males were tested for their aggressive behavior; biting males were excluded. To avoid habituation, mice were replaced to a new cage with a new dominant mouse after 7 and 14 days, respectively. In parallel, control mice were single-housed (SHC) for the indicated times. CSC and SHC mice were taken into experiments on day 20, i.e. directly after 19 days of CSC/SHC, or 9 or 23 days after termination of CSC/SHC. As a matter of routine, the subordinate position of each CSC mouse was confirmed by behavioral analysis of the first 30 min after setting up the CSC colonies.

### Cells

Single cells suspensions from spleen and bone marrow of femurs were prepared. Spleens were carefully passed through a 40 μm cell strainer (BD Biosciences, Heidelberg, Germany), washed twice with PBS/2% FCS and erythrocytes were lysed with hypertonic solution (0.17 M NH4Cl with 20 mM HEPES in H_2_O) for 10 min at 37°C. For isolation of bone marrow cells epicondyles were cut and diaphyses were flushed with 5 ml 1x PBS. Cells were counted with a haemocytometer (A.Hartenstein, Würzburg, Germany). Peripheral blood for FACS-staining was isolated with EDTA-coated microvettes (Sarstedt, Nümbrecht, Germany).

The BFS1 fibrosarcoma cells were generated in a female C57BL/6N mouse by injection of 1 mg of 3-methylcholanthrene (Sigma, Taufkirchen, Germany) dissolved in 200 μl tricaprylin (Sigma) i.d. in the back of a mouse as described [35]. Syngeneic tumor cells (BFS1, 2x10^5^ in 50μl PBS) were subcutaneously implanted on the upper part of the loin. The diameters of the tumors were measured in two directions perpendicularly to each other with a caliper.

### Chemical sympathectomy

Chemical sympathectomy was performed as described [36]. Animals were treated with an intra-peritoneal injection of 6-hydroxy-dopamine (6OHDA, Sigma, Taufkirchen, Germany) in 0.1% ascorbic acid in PBS (80 mg/kg) on days -8, -7, and -6 before initiation of CSC/SHC. To maintain sympathectomy, animals were treated with a fourth intraperitoneally injection of 6OHDA on day 15 of CSC. Control animals were treated with 0.1% ascorbic acid in PBS.

### MDSC isolation

CD11b^+^ cells were isolated from spleen and bone marrow cells by magnetic separation with a negative selection antibody cocktail (containing B220-PE, CD3-PE, CD4-PE, CD8-PE, CD11c-PE, NK1.1-PE and cKit-PE) and PE-positive cells were depleted with anti-PE MicroBeads according to the manufacturer’s instructions (Miltenyi Biotec GmbH, Bergisch Gladbach, Germany). Ly6G^+^ cells were positively selected with magnetic beads (Miltenyi Biotec). The resulting cells were CD11b^+^Ly6C^int^Ly6G^+^ (PMN-MDSC, >95% purity); the negative population was mainly CD11b^+^Ly6C^+^Ly6G^-^ (MO-MDSC, up to 65% purity) with contaminating PMN-MDSC.

### Cell stimulation *in vitro*

Splenocytes or MDSC were cultured in RPMI containing 10% FCS, Penicillin, Streptomycin, 50μM β-mercaptoethanol (PAN Biotech, Aidenbach, Germany) and stimulated with LPS (E.coli; Serotyp: O111:B4, 100 ng/ml, Sigma-Aldrich) and mouse interferon (IFN)γ (120-240U/ml) for 72 h. Supernatants were harvested and stored at -20°C for further analysis.

ELISA detection kits for tumor necrosis factor (TNF), Interleukin (IL)-6, IFNγ and IL-10 were used for quantification of cytokine levels in supernatants (R&D Systems, Wiesbaden, Germany). Nitrite concentrations were determined using Griess reagent measuring the optical density at 540 nm.

### Flow cytometry

Splenocytes or bone marrow cells (10^6^) were washed with PBS/ 2% FCS, cell pellets were re-suspended in blocking solution (10% mouse serum, 10% FCS in PBS), and labelled with fluorochrome- or biotin-labelled antibodies. For phenotypic analysis the following monoclonal antibodies were used: CD11b-PE (M1/70), CD11b-APC (M1/70), B220-PerCP (RA3-6B2), B220-biotin (RA3-6B2), MHCII-AF700 (M5/114.15.2), Gr1-eF660 (RB6-8C5), CD4-PE (RM4-5) and CD11c-biotin (N 418) purchased from eBioscience (Frankfurt, Germany); CD11c-AF700 (HL3), Gr1-FITC (RB6-8C5), CD8-PE (53–6.7), CD11c-PE (HL3), cKit-PE (ACK45), Ly6C-biotin (AL-21), Ly6C-FITC (AL-21) and Ly6G-V450 (1A8) obtained from BD Biosciences (Heidelberg, Germany), MHCII-APC (M5/114.15.2) from Miltenyi Biotec GmbH (Bergisch Gladbach, Germany), CD3-PE (17A2) from BioLegend (London, United Kingdom), and CD11b-biotin (M1/70) from Fitzgerald (Concord, USA). Streptavidin-Pacific Orange and Streptavidin-AF546 conjugates (Life Technologies; Darmstadt, Germany) were used as secondary reagents to detect biotinylated antibodies. The intracellular staining procedure for Treg cell detection was performed by using the Foxp3-PE antibodies and the Foxp3 staining buffer set from eBiosciences. For nuclear staining DAPI (Sigma-Aldrich, Taufkirchen, Germany) was used. Following incubation of primary antibodies for 30 min at 4°C in PBS/2% FCS, leucocytes were washed with PBS/2% FCS. In case biotinylated primary antibodies were used, a secondary streptavidin-Pacific Orange conjugate was dissolved in PBS/2% FCS and incubated 30min with the samples at 4°C. After washing coupled antibodies were fixed with PBS/2% paraformaldehyde. Whole blood cell staining was performed using FACS Lysing solution (BD Biosciences). Cells were measured on a BD LSR-II cytometer and analyzed using FACSDiva software (BD Biosciences). Living single cells were gated based on FSC/SSC properties and lymphoid and myeloid cell populations were gated according to their expression of surface or intracellular markers.

### T cell suppression assay

To obtain different ratios of MDSC to responder T cells, MDSC were cultured at increasing numbers in 100 μl RPMI in 96-well round bottom plates overnight before addition of 100 μl RPMI with 2x10^5^ 5,6-carboxyfluorescein succinimidyl ester (CFSE, eBioscience)-labeled splenocytes as responder cells. Cells were stimulated for three days with soluble anti-CD3e-antibodies (1 μg/ml, BD Pharmingen) and anti-CD28-antibody (0.5 μg/ml, eBioscience) and analyzed by flow cytometry. Living single cells were gated based on FSC/SSC properties and their expression of CD4 or CD8. Proliferation was determined according to their CFSE profile.

### Immunohistochemistry

Spleens and tumors were cut into 8 μm slices from the central part of spleens or tumors, respectively, put on a microscope slide (Thermo Scientific, Braunschweig, Germany) and dried at room temperature overnight. Specimens were fixed with Acetone (-20°C, 10 min), washed with PBS/0.05% Tween20 and unspecific binding was blocked with PBS/10% mouse serum (v/v). Splenic samples were stained with primary mAbs (CD11b-APC, Gr1-FITC, B220-biotin, eBioscience) diluted in PBS/10% FCS, washed and the secondary streptavidin-AF546 conjugate solution (Life Technologies) was applied. Tumor tissues were stained with primary mABs (Gr1-FITC and Meca32-biotin, BD Bioscience) diluted in PBS/10% FCS, washed and secondary streptavidin-AF546 conjugate solution was applied. To stain CD31^+^ cells in the tumor, specimens were stained with rat-anti CD31 mAB (BD Bioscience) diluted in PBS/10% FCS, washed and secondary antibody (goat-anti-rat AF647, Life Technologies) was applied. After washing slides were mounted with Perma Fluor (Thermo Scientific, Braunschweig, Germany) and analyzed Axio Imager fluorescent microscope (Carl Zeiss, Jena, Germany) Quantification of CD31 expression was performed using the TissueFAXS system (TissueGnostics GmbH, Vienna, Austria).

### Statistics

Unpaired Student´s t-test or two-way ANOVA with Bonferroni post hoc test were used in experiments with two or more experimental groups. P<0.05 was accepted as significantly different. Statistics were performed using GraphPad Prism 5.0 (GraphPad Software, La Jolla, CA, USA). All data shown in figures are representative of at least 2–3 independent experiments until stated otherwise.

## Results

### Accumulation of myeloid cells during chronic stress

**Fig 1:**
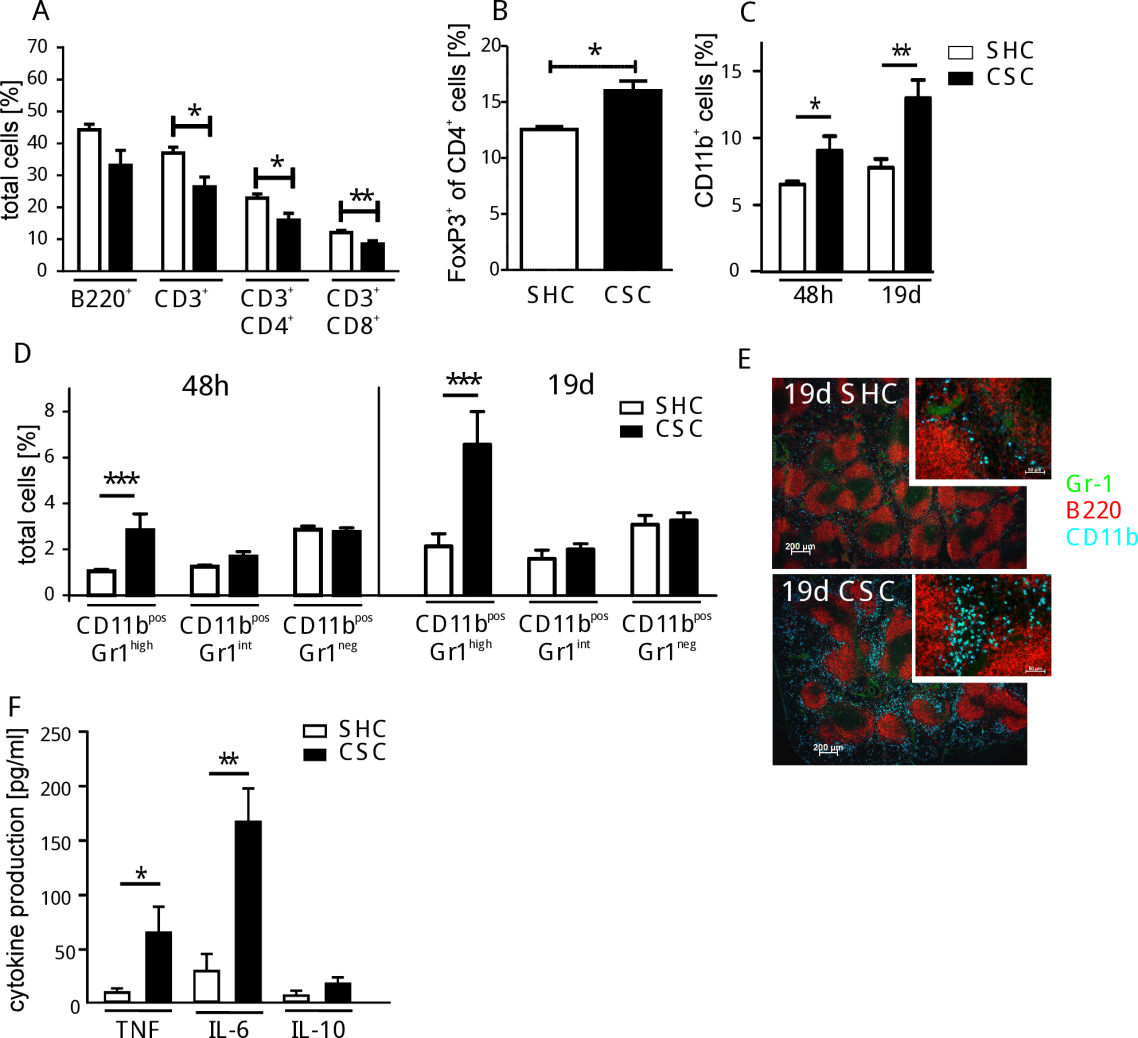
Effect of CSC on spleen cells. Splenocytes from CSC (black bars) and SHC mice (white bars) were isolated and analyzed by flow cytometry (total splenocytes SHC, n = 7: 4.2±1.7x10^7^; CSC, n = 8: 9.8±2.4x10^7^). The percentage of splenic B (SHC: 1.9±0.8x10^7^; CSC: 3.2±0.8x10^7^) and T cells (SHC: 1.6±0.3x10^7^; CSC: 2.6±0.6x10^7^) (**A)** and Treg cells (SHC: 2.0±0.4x10^6^; CSC: 2.6±0.6x10^6^) **(B)** were quantified after 19d of SHC/CSC. Splenic CD11b^+^ cells **(C)** and CD11b^+^ cells differentiated by their Gr1 expression level were analyzed after 48h and 19d of CSC (SHC: n = 8, CSC: n = 8) **(D)**. Immunohistochemical staining of spleen sections from mice after 19d of CSC/SHC for CD11b^+^ (blue), Gr1^+^ (green), and B220^+^ (red) cells was performed **(E)**. Splenocytes from individual mice after 19d CSC/SHC were stimulated with LPS followed by determination of TNF, IL-6, and IL-10 levels in the supernatants (SHC: n = 4, CSC: n = 4) **(F)**. *p < 0.05; **p < 0.01; ***p < 0.001 (Student´s t-test). These experiments were performed at least 3 times.

Social stress has recently been shown to up-regulate myelopiesis and mediate immune activation in humans and mice [5]. In order to determine how CSC affects the immune system in naïve mice, the spleen cell composition was compared after 19 days of CSC or SHC, respectively. Due to the CSC-induced increased cellularity of the spleen in the experiments shown here, a relative contraction of the lymphocyte compartment (from 80.9% to 59.2%) was observed in the spleen (Fig 1A), paralleled by an expansion of the Foxp3^+^ Treg cell fraction in the CD4^+^ T cell population (from 2.0±0.4x10^6^ to 2.6±0.6x10^6^ cells) (Fig 1B). The myeloid cell compartment in the spleen increased strongly during CSC (from 3.5±0.6x10^6^ to 5.1±2.0x10^6^ cells after 48h and from 3.4±0.6x10^6^ to 6.9±2.7x10^6^ cells after 19d). An increased proportion of CD11b^+^ cells was detectable already 48 hours after the onset of CSC and this expansion of CD11b^+^ cells was even more pronounced after 19 days of CSC compared to SHC mice (Fig 1C). To examine the phenotype of these CSC-induced myeloid splenocytes in more detail, flow cytometric analysis was performed using the myeloid cell specific markers CD11b, CD11c, Gr1, Ly6C, Ly6G, and MHCII (S1 Fig). Based on scatter characteristics and high intensity of Gr1 expression, we identified the CSC-induced increase in CD11b^+^ cells after 48h and 19d (Fig 1D) as granulocytic immature myeloid cells with uniformly polymorphonuclear shape. Histological analysis revealed Gr1^+^ myeloid (CD11b^+^) cells accumulating preferentially in the red pulp of the spleen clustering around B cell follicles (Fig 1E). Pro-inflammatory cytokines, such as TNF and IL-6, produced by stimulated splenocytes were found to be increased by CSC, while the production of anti-inflammatory IL-10 was not affected (Fig 1F), clearly supporting the previously reported immune-stimulatory effects of CSC [13].

**Fig 2:**
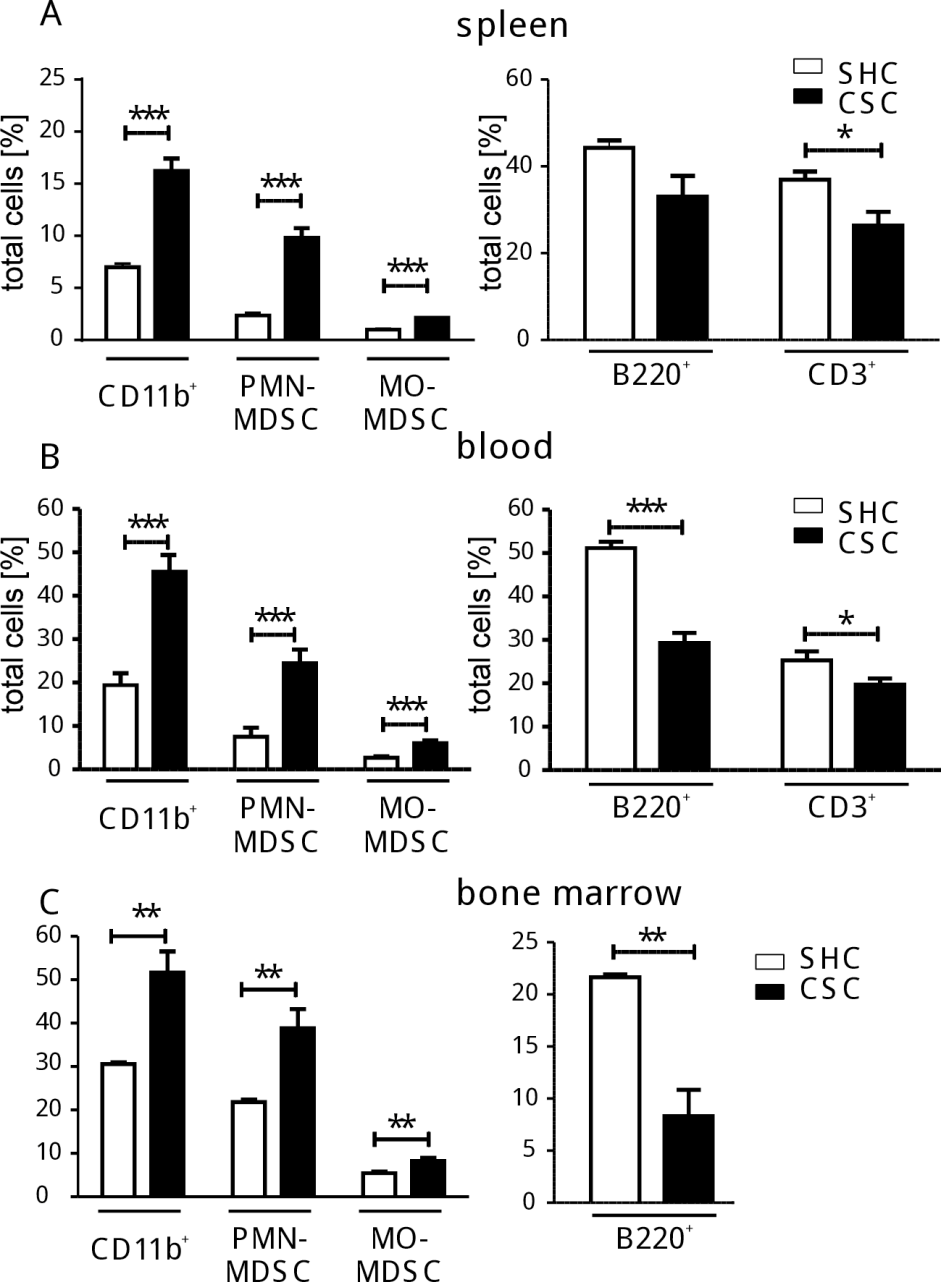
Induction of immature myeloid cells by CSC. Splenocytes from CSC (black bars) and SHC (white bars) mice were isolated and analyzed by flow cytometry. The percentages of splenocytes from individual mice (SHC: n = 4, spleen cell pool: 1.7x10^8^; CSC: n = 4, spleen cell pool: 2.4x10^8^) **(A)**, of blood cells counted in 0.1ml of blood (SHC: n = 8, CSC: n = 8) **(B),** and of bone marrow cells (SHC: n = 3, CSC: n = 3) **(C)** are shown depending on their staining pattern for CD11b^+^Ly6G^+^Ly6C^int^ (PMN-MDSC), and CD11b^+^Ly6G^-^Ly6C^high^ (MO-MDSC) (left graphs) or B220^+^ and CD3^+^ (right graphs). *p < 0.05; **p < 0.01; ***p < 0.001 (Student´s t-test). These experiments were performed more than 3 times.

Stratification of CD11b^+^ cells according to their expression of Ly6C and Ly6G (S2 Fig) revealed enhanced proportions of cells with either granulocytic (Ly6C^int^ Ly6G^+^) or monocytic (Ly6C^high^ Ly6G^-^) phenotype corresponding to the so-called PMN-MDSC or MO-MDSC, respectively, in the spleens of CSC mice (Fig 2A, left graph). The lymphocyte compartment seemed relatively contracted in CSC mice, due to the increased frequency of myeloid cells (the myeloid cell fraction increased from 1.7% to 4.1% while lymphocytic fraction only increased from 20.6% to 24.2% after CSC) (Fig 2A, right graph). This CSC-induced shift was not restricted to the spleen cells but was also observed in blood (Fig 2B) and bone marrow (Fig 2C) indicating an increased myelopoietic output and cellular shift in the immune system.

### TNF and catecholaminergic signaling contribute to CSC-induced accumulation of myeloid cells

**Fig 3:**
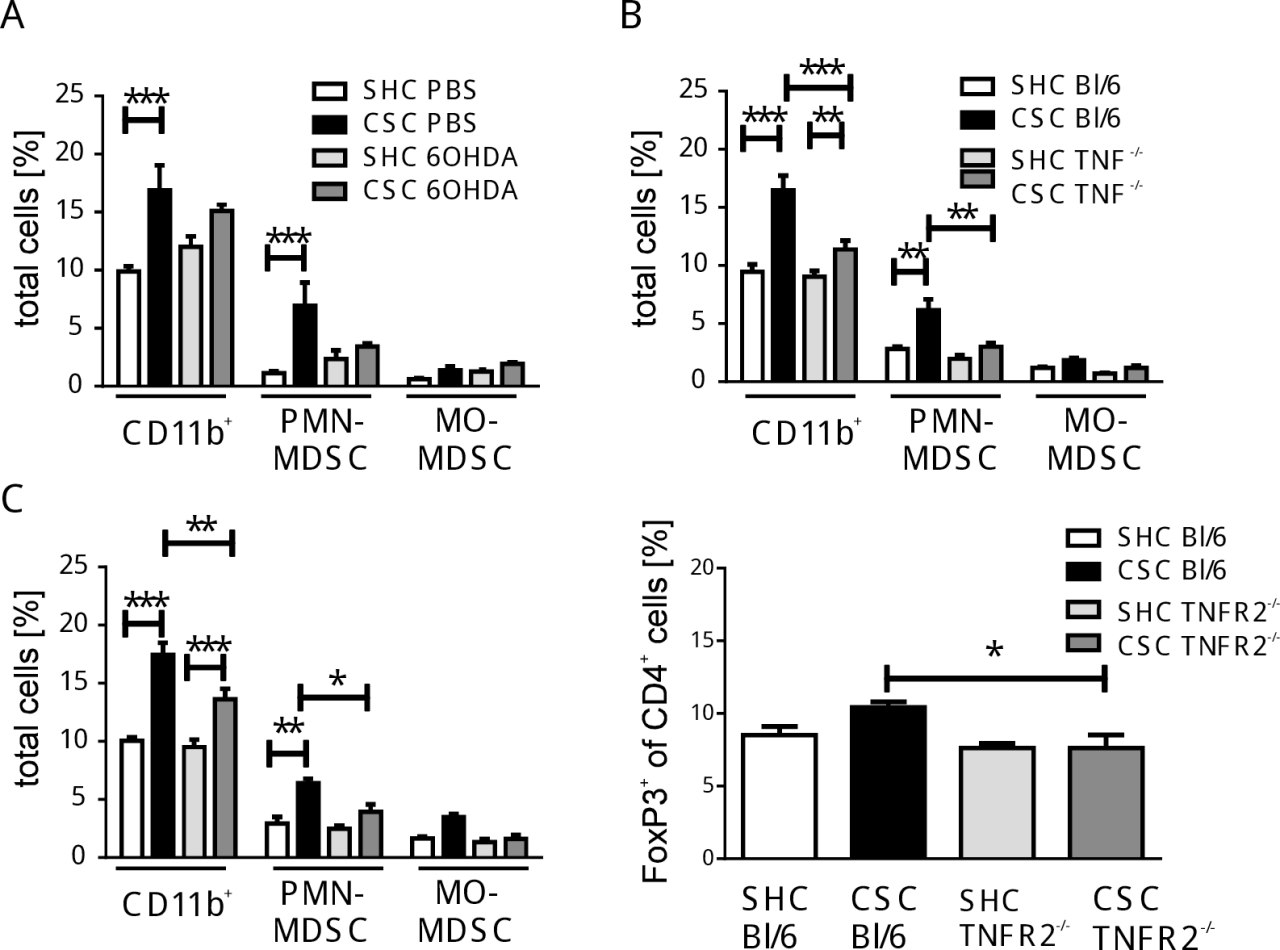
Involvement of catecholamines, TNF, or TNFR2 on CSC-caused cellular shifts. **(A)** Mice were treated either with PBS or 6-OH dopamine (6OHDA) before induction of CSC/SHC. Splenocytes from individual PBS-treated SHC (white bars, n = 4, total splenocytes: 3.4±0.6x10^7^) and CSC mice (black bars, n = 3, total splenocytes: 4.1±1.6x10^7^), 6OHDA-treated SHC (light grey bars, n = 4, total splenocytes: 4.1±0.7x10^7^) and CSC mice (dark grey bars, n = 3, total splenocytes: 6.3±1x10^7^) were isolated after 19d of SHC/CSC and analyzed by flow cytometry. The percentages of CD11b^+^ cells (SHC/PBS: 3.3±0.6x10^6^; CSC/PBS: 6.9±2.7x10^6^; SHC/6OHDA: 4.9±0.8x10^6^; CSC/6OHDA: 9.5±0.2x10^6^), CD11b^+^Ly6G^+^Ly6C^int^ (PMN-MDSC, SHC/PBS: 0.4±0.1x10^6^; CSC/PBS: 2.9±1.1x10^6^; SHC/6OHDA: 1.0±0.2x10^6^; CSC/6OHDA: 2.1±0.0x10^6^), and CD11b^+^Ly6G^-^Ly6C^high^ (MO-MDSC, SHC/PBS: 0.2±0.03x10^6^; CSC/PBS: 0.6±0.2x10^6^; SHC/6OHDA: 0.5±0.1x10^6^; CSC/6OHDA: 1.2±0.02x10^6^) were determined. **(B)** Wild type (Bl/6) or TNF-deficient (TNF^-/-^) mice were exposed to SHC/CSC. The percentages of CD11b^+^ cells, PMN-MDSC, and MO-MDSC of individual Bl/6-SHC- (white bars, n = 4, total splenocytes: 4.2±1.7x10^7^), Bl/6-CSC- (black bars, n = 3, total splenocytes: 8.9±2.4x10^7^), TNF^-/—^SHC (light gray bars, n = 6, total splenocytes: 6.9±1.3x10^7^), and TNF^-/—^CSC-mice (dark grey bars, n = 5, total splenocytes: 9.3±1.9x10^7^) were quantified. The percentages of CD11b^+^ cells (SHC/Bl/6: 4.0±1.6x10^6^; CSC/Bl/6: 16.1±4.0x10^6^; SHC/TNF^-/-^: 6.4±1.4x10^6^; CSC/TNF^-/-^: 10.8±2.4x10^6^), CD11b^+^Ly6G^+^Ly6C^int^ (PMN-MDSC, SHC/Bl/6: 1.2±0.5x10^6^; CSC/Bl/6: 6.0±1.5x10^6^; SHC/TNF^-/-^: 1.4±0.3x10^6^; CSC/TNF^-/-^: 2.9±0.1x10^6^), and CD11b^+^Ly6G^-^Ly6C^high^ (MO-MDSC, SHC/Bl/6: 0.5±0.3x10^6^; CSC/Bl/6: 1.8±0.5x10^6^; SHC/TNF^-/-^: 0.5±0.1x10^6^; CSC/TNF^-/-^: 1.1±0.2x10^6^) were determined. **(C)** Wild type (Bl/6-SHC, n = 3, white bars, total splenocytes: 7.1±1.5x10^7^; Bl/6-CSC, n = 4, black bars, total splenocytes: 9.5±2.1x10^7^) or TNFR2-deficient (TNFR2^-/-^ SHC, n = 4, light gray bars, total splenocytes: 6.3±1.2x10^7^; TNFR2^-/-^ CSC, n = 4, dark grey bars, total splenocytes: 5.1±0.9x10^7^) mice were exposed to SHC/CSC. The percentages of CD11b^+^ cells (SHC/WT: 7.1±1.5x10^6^; CSC/WT: 17.8±3.9x10^6^; SHC/TNFR2^-/-^: 5.9±1.1x10^6^; CSC/TNFR2^-/-^: 6.9±1.2x10^6^), PMN-MDSC (SHC/WT: 2.0±0.4x10^6^; CSC/WT: 6.1±1.3x10^6^; SHC/TNFR2^-/-^: 1.6±0.3x10^6^; CSC/TNFR2^-/-^: 2.0±0.4x10^6^), and MO-MDSC (SHC/WT: 1.2±0.2x10^6^; CSC/WT: 3.3+0.7x10^6^; SHC/TNFR2^-/-^: 0.9±0.2x10^6^; CSC/TNFR2^-/-^: 0.8±0.14x10^6^)(left graph) or Treg cells (SHC/WT: 1.4±0.2x10^6^; CSC/WT: 2.0±0.4x10^3^; SHC/TNFR2^-/-^: 1.0±0.2x10^6^; CSC/TNFR2^-/-^: 0.6±0.1x10^6^)(right graph) were quantified. These experiments were performed twice. *p < 0.05; **p < 0.01; ***p < 0.001 (2way ANOVA).

Based on the above mentioned animal data suggesting a prominent role for the SNS in chronic stress-induced myelopoiesis and migration of myeloid cells into the periphery [9;10;37], chemical sympathectomy induced by intraperitoneal injections of 6-hydroxy-dopamine (6OHDA) [38] before the start of CSC was applied to test whether CSC-induced cell shifts would be affected. The splenic accumulation of myeloid cells and particularly PMN-MDSC was clearly less pronounced compared to controls without sympathectomy (Fig 3A) while the lymphocyte compartment seemed not affected, indicating catecholaminergic sensitivity of especially the immature myeloid cells.

The enhanced capacity of splenocytes for inflammatory cytokine production after 19d of CSC (Fig 1E) pointed to a possible role of inflammatory cytokines in the CSC-induced cellular shifts. In particular, TNF has been found to support generation of Treg cells as well as MDSC by activation of TNF receptor type 2 (TNFR2) [39–44]. Therefore, the spleen cell composition in TNF- and TNFR2-deficient mice was compared to wild type mice after 19d of CSC or SHC, respectively. Although CSC caused similar cellular changes in these mouse lines as in wild type mice, those cellular shifts were significantly less pronounced in mice with a genetic ablation of either TNF (Fig 3B, CD11b^+^, PMN-MDSC) or TNFR2 (Fig 3C, left graph, CD11b^+^, PMN-MDSC). The reduced CSC-induced cellular shift in TNFR2-deficient mice was also seen in the bone marrow and the blood [45]. In addition, in this mouse line the proportion of splenic Foxp3-positive Treg cells in the CD4^+^ T cell population was significantly lower after CSC compared to respective wild type mice (Fig 3C, right graph). These data indicate that the generation of suppressive cells types after CSC, in particular MDSC, partly depends on activation of TNFR2.

### Functional activity of myeloid cells after CSC

**Fig 4:**
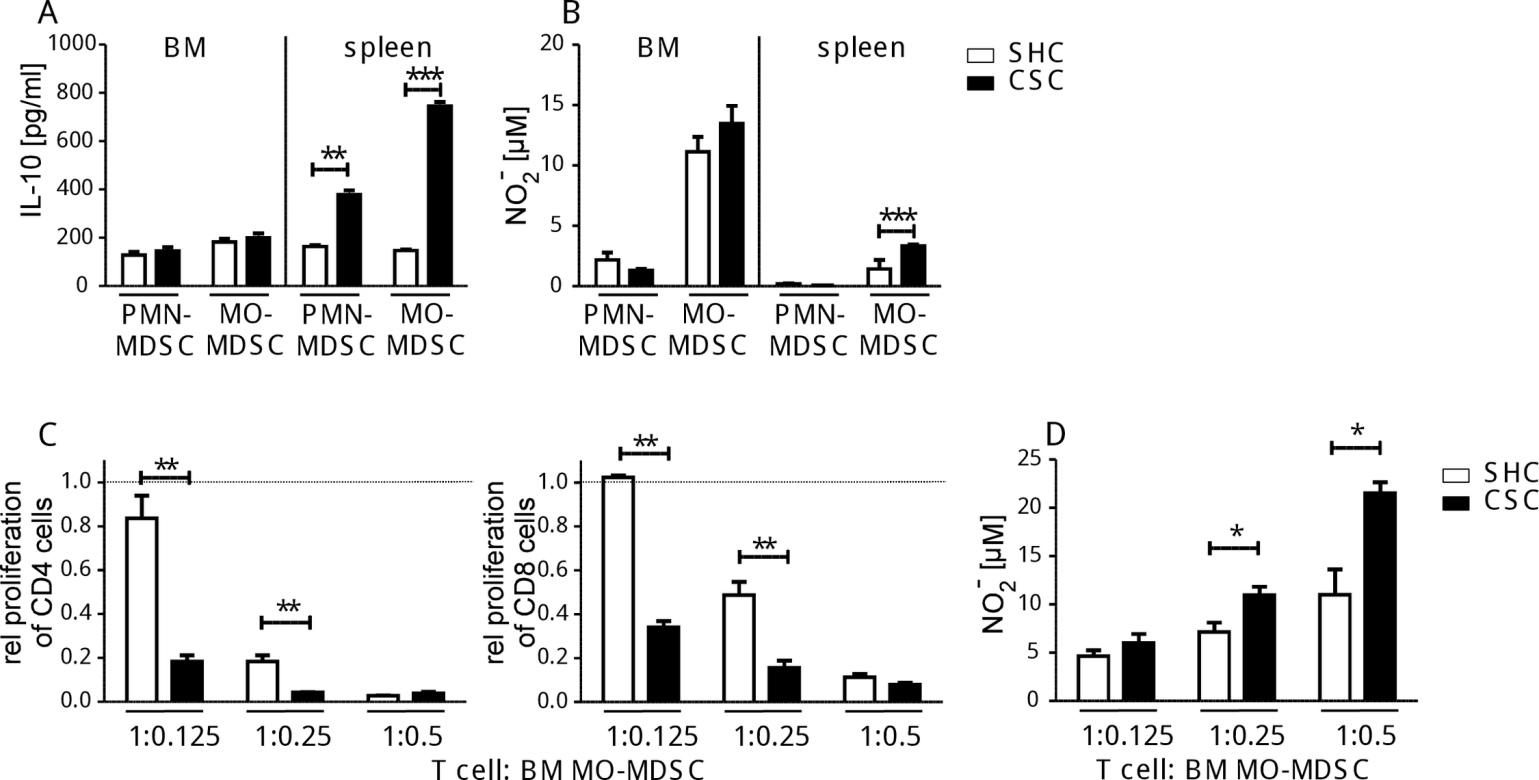
Functional activity of bone marrow-derived PMN-MDSC and MO-MDSC after CSC. CD11b^+^Ly6G^+^Ly6C^int^ cells (PMN-MDSC) and CD11b^+^Ly6G^-^Ly6C^+^ cells (MO-MDSC) were isolated from bone marrow (BM) or spleen of individual mice after 19d of CSC (black bars, n = 4) or SHC (white bars, n = 4) and stimulated with LPS + IFNγ followed by IL-10 **(A)** and NO_2_^-^ **(B)** determination in the supernatants. MO-MDSC (CD11b^+^Ly6G^-^Ly6C^+^) were isolated from the bone marrow of mice after 19d of CSC (black bars, n = 4) or SHC (white bars, n = 4) and co-cultured with proliferating splenocytes from naive mice. Relative proliferation of CD4^+^ (left graph) and CD8^+^ (right graph) T cells is shown at different T cell to MO-MDSC ratios **(C).** The NO_2_^-^ contents of the supernatants from (C) were measured **(D)**. These experiments were performed once *p < 0.05; **p < 0.01; ***p < 0.001 (Student´s t-test).

Bone marrow and spleen cells of CSC and SHC mice were isolated as PMN-MDSC and MO-MDSC preparations, respectively, according to their expression of CD11b, Ly6C and Ly6G. The *in vitro* anti-inflammatory and suppressive capacity of these myeloid subpopulations was tested by assessing their IL-10 and NO_2_^-^ release during LPS stimulation (Fig 4A and 4B) as well as their suppressive effects in a T cell proliferation assay (Fig 4C). In detail, splenic MDSC isolated from CSC mice produced more IL-10 (PMN-MDSC and MO-MDSC, Fig 4A) and NO_2_^-^ (only MO-MDSC, Fig 4B). Bone marrow-derived MO-MDSC from CSC mice inhibited proliferation of CD4^+^ and CD8^+^ T cells significantly stronger than respective cells from SHC mice (Fig 4C). This was paralleled by increased NO_2_^-^ concentrations in the supernatants of those proliferation assays containing MO-MDSC at least at T cell to MO-MDSC ratios of 1:0.25 and 1:0.5 (Fig 4D). In contrast, splenic MO-MDSC isolated after 19d of CSC did not suppress CD4^+^ and/or CD8^+^ T cell proliferation irrespective of previous CSC even at the highest MDSC to T cell ratio (2 to 1) tested, matching their relatively low NO_2_^-^ production capacity (Fig 4B) compared to MO-MDSC isolated from the bone marrow.

### Tumor growth after CSC

**Fig 5:**
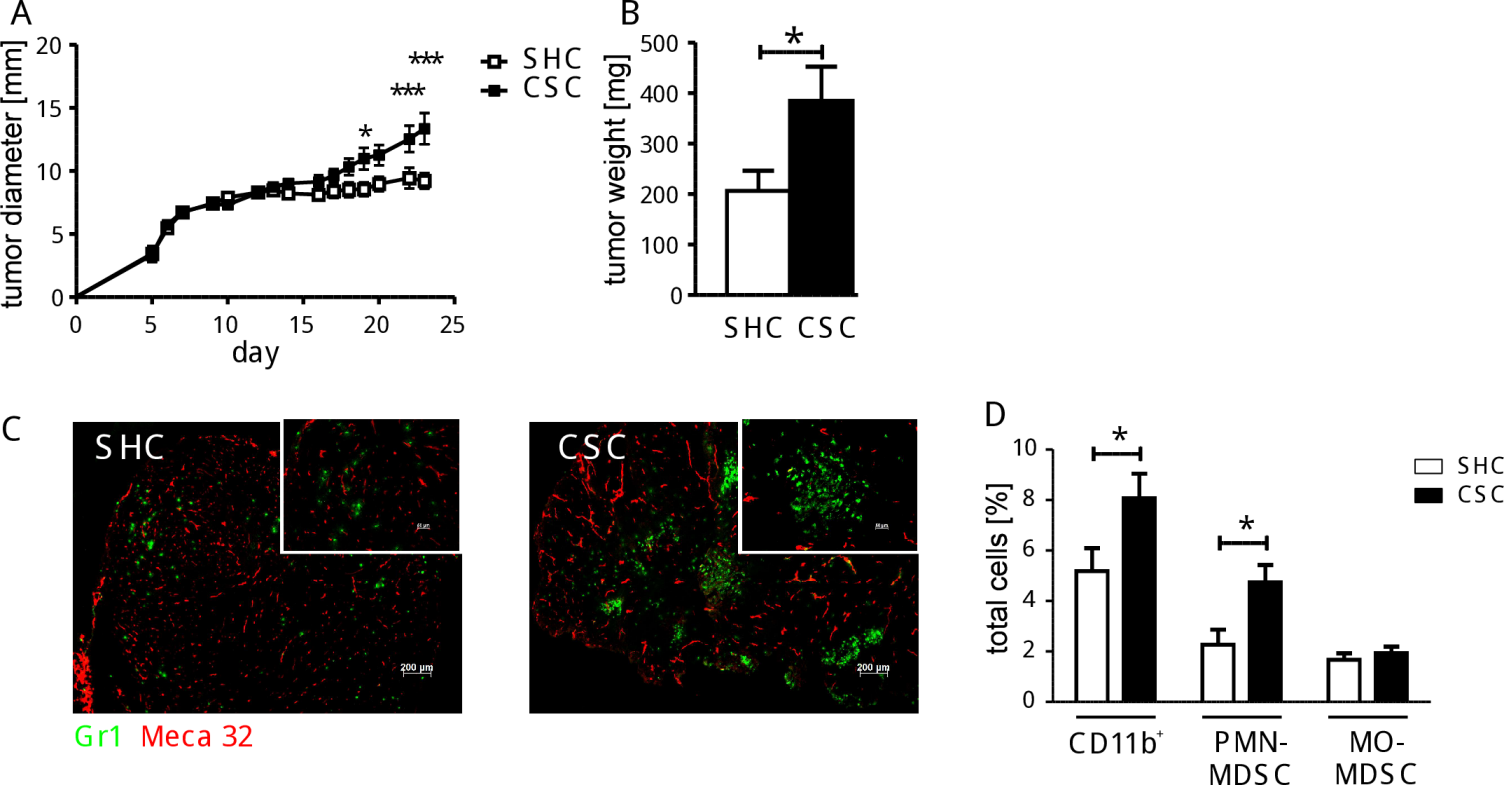
Effect of CSC after 23 days of tumor growth. BFS1 tumor cells were inoculated subcutaneously after 19 days of either SHC (white bars, n = 7) or CSC (black bars, n = 8) and tumor growth was monitored for 23 days **(A). T**he weight of the tumor tissue was determined on day 23 after termination of CSC/SHC **(B)**. Immunohistochemical staining is shown for Gr1^+^ (green) and endothelial (Meca32, red) cells in tumor sections **(C)**. Percentages of CD11b^+^cells, CD11b^+^Ly6G^+^Ly6C^int^ cells (PMN-MDSC), and CD11b^+^Ly6G^-^Ly6C^high^ cells (MO-MDSC) in spleens of individual mice were quantified (SHC: n = 7, total splenocytes: 4.9±1.7x10^7^, CSC: n = 8, total splenocytes: 5.6±0.7x10^7^) **(D)**. This experiment was performed once *p < 0.05; ***p < 0.001 (Student´s t-test).

To investigate the consequences of these CSC-induced cellular changes, syngeneic fibrosarcoma cells were subcutaneously transplanted into mice after 19 days of SHC/CSC exposure in order to compare tumor growth. Several days after implantation tumors started to grow and from day 9 after implantation on it became visible that tumors from mice previously exposed to CSC grew better compared to those implanted in SHC mice. CSC mice compared to SHC mice had significantly larger tumors with more tumor mass after 23 days of tumor growth (Fig 5A and 5B). Histology of the solid tumors from CSC mice revealed stronger vascularization, indicated by a higher density of endothelial cell marker molecules stained by Meca-32, and the presence of larger numbers of Gr1-positive immature myeloid cells compared to SHC mice (Fig 5C).

At this time point—23 days after termination of CSC/SHC—spleens from tumor bearing CSC mice had a higher proportion of myeloid cells than respective SHC mice (Fig 5D). Subtyping of the CD11b^+^ cell population showed that only the PMN-MDSC, but not the MO-MDSC fraction, was significantly higher in tumor bearing CSC mice compared to respective cell fractions from SHC mice.

### Early tumor-dependent changes after CSC

**Fig 6:**
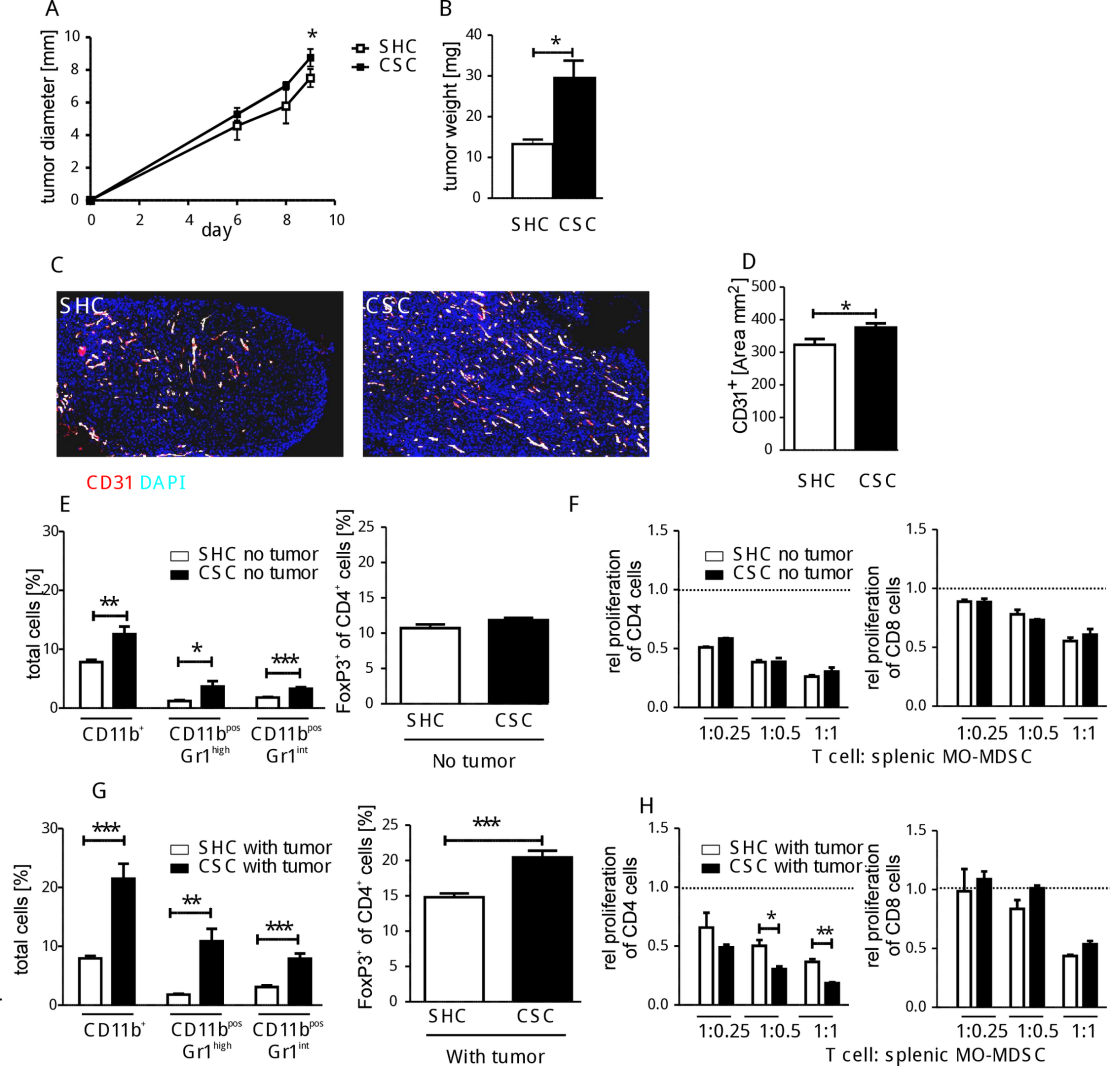
Effect of CSC after 9 days of tumor growth. BFS1 tumor cells were inoculated subcutaneously after 19 days of either SHC (white bars, n = 7) or CSC (black bars, n = 8) and tumor growth was monitored for 9 days **(A)** and the tumor weight was determined **(B)**. Immunohistochemical staining is shown for vessels (CD31, red) and cell nuclei (DAPI, blue) in 6 central tumor sections per mouse (magnification 50-fold) **(C)**. The area of CD31^+^ cells in tumor sections from mice was quantified after SHC/CSC **(D)**. Spleen cells from tumor-free **(E, F)** and of tumor-bearing mice **(G, H)** 9 days after termination of CSC/SHC were analyzed. Percentages of CD11b^+^ cells, CD11b^+^Gr1^high^, CD11b^+^Gr1^int^ cells and Treg cells were determined in spleens of individual tumor-free (total splenocytes plus CD4 cells without (w/o) tumor CSC/SHC **(E, left graph)** or tumor-bearing CSC/SHC-mice (total splenocytes plus CD4 cells with tumor CSC/SHC **(G, left graph)**. CD11b^+^Ly6G^-^Ly6C^+^ cells (MO-MDSC) were isolated from spleens of individual tumor-free **(F)** or tumor-bearing-SHC/CSC-mice **(H)** and co-cultured with proliferating splenocytes from naive mice. Relative proliferation of CD4^+^ (left graphs) and CD8^+^ (right graphs) T cells is shown at different T cell to MDSC ratios. This experiment was performed once *p < 0.05; **p < 0.01 (Student´s t-test).

Since transplanted tumor cells depend on vascularization for continuous tumor growth, we assessed whether the enhanced tumor growth in CSC mice is paralleled by an improved vascularization detectable 9 days after transplantation. In line with what we reported for day 23 after tumor cell transplantation, tumor size and weight started to be enhanced in CSC compared with SHC mice 9 days after implantation (Fig 6A and 6B). In these very small tumors, enhanced vascularization in CSC mice was revealed by histology compared to the SHC mice using CD31 as an endothelial marker (Fig 6C and 6D).

Analysis of spleen cells from the tumor-bearing mice at this early time point of tumor growth indicated, that the CSC-induced shift towards increased immature myeloid cells was still detectable in both tumor-free (Fig 6E, left panel) and tumor-bearing mice (Fig 6G, left panel) as reported directly after 19 days of CSC. Notably, this CSC-induced shift seemed to be more pronounced in tumor-bearing (Fig 6G) compared to tumor-free mice (Fig 6E), as indicated by a CSC-induced increase in CD11b^+^, PMN-MDSC, and MO-MDSC cells (13.5%, 9%, and 4.8% in tumor-bearing versus 4.8%, 2.5%, and 1.5% in tumor-free mice). Interestingly and in contrast to tumor-free mice (Fig 6E, right graph), tumor-bearing CSC-mice 9 days after termination of CSC/SHC still showed an increased Treg cell proportion in the CD4^+^ T cell population (Fig 6G, right graph) similar to what has been observed directly after termination of CSC (Fig 1B). The finding that the CSC-induced increase in the Treg cell frequency was not detectable any more in mice without tumor implantation 9 days after termination of CSC (Fig 6E, right graph) demonstrated that the CSC-induced increase in Treg frequency shown in Fig 1B was transient. The strong increase in the frequency of Treg cells in tumor-bearing CSC mice (20.4±0.9%) compared to the frequency found in the respective tumor-free CSC mice (11.8±1.0%) at this time point suggested that tumor transplantation maintained or even aggravated the CSC induced effects. Splenic MO-MDSC preparations from tumor-free CSC and SHC mice suppressed T cell proliferation to a similar extent (Fig 6F). This is in line with the observation that no CSC-enhanced T cell suppression was observed right after termination of CSC/SHC [44]. Surprisingly and in contrast to the time directly after termination of CSC, splenic MO-MDSC from tumor-bearing CSC mice 9 days after termination of CSC were now more suppressive for CD4^+^ T cells (Fig 6H, left) when compared to respective MO-MDSC from SHC mice. The suppressive activities of CSC- and SHC-derived splenic MO-MDSC against CD8^+^ T cells were comparable. These data demonstrate a tumor-induced gain of suppressive activity towards CD4^+^ T cells by splenic MO-MDSC which was more pronounced by splenic CSC- versus SHC-MO-MDSC.

## Discussion

In the present study we demonstrated that chronic psychosocial stress in mice results in pronounced activation of innate immunity in the spleen, indicated by an increased LPS-induced pro-inflammatory cytokine secretion by isolated splenocytes. In parallel, numbers of MDSC and Treg cells were enhanced and in case of MDSC also their suppressive capacity. Catecholaminergic and TNF signaling were found to be relevant for these CSC-induced changes. Moreover, increased occurrence and functionality of suppressor cell types were accompanied by enhanced growth of transplanted syngeneic fibrosarcoma cells, more pronounced angiogenesis, and increase of MDSC in tumor tissue.

Acute stress induces physiological and immunological changes preparing the organism to cope with invading pathogens and possible tissue damage. The acute stress response, therefore, is associated with a transient immune activation, immediately counter-regulated by the HPA axis, the SNS, and the immune system to redress the balance towards homeostasis. The latter is a prerequisite for adequate immune responses upon upcoming challenges. Chronic stress, in contrast, has been shown to disturb homeostasis, resulting in either immune suppression [1] or over-activation of the immune system [5;6]. In addition, an enhanced risk for cancer development has been reported to be associated with chronic stress by several authors [7;8]. Employing the CSC mouse model, we showed earlier that chronic psychosocial stress promotes activation and differentiation of T cells into Th1, Th2, and Th17 effector cells in peripheral lymph nodes, although contracting the T cell compartment with respect to the overall cell number [13]. An increased inflammatory status in CSC versus SHC mice has been further documented by the development of spontaneous colitis during CSC exposure [12]. This was indicated by an increased histological damage score and cytokine secretion from *ex vivo*-stimulated mesenterial lymph node cells derived from CSC mice [12;45]. An increased capacity to produce inflammatory cytokines by splenocytes from mice has also been observed in another mouse model of chronic psychosocial stress [9]. Together with an increased number of germinal centers in the spleen of CSC versus SHC mice [13], these data clearly demonstrate the activation of cells in secondary lymphoid organs. Interestingly, the expanded Treg cell fraction in the CD4^+^ population after CSC, as shown in the current study, suggests that activation of adaptive immunity is accompanied by generation of regulatory T cells, presumably as counter-regulatory approach to dampen stress-induced immune activation. This is supported by findings showing reciprocal stimulation of both Th17 and Treg cells [46]. Notably, an enhanced frequency and suppressive function of Treg cells has also been shown following chronic restraint stress [47].

Data of the current study further suggest that T cell inhibition, or counter-regulation of adaptive immunity in general, might also be mediated by expansion of T cell suppressive MDSC in CSC mice. Although not studied here in detail, MDSC have been reported to show potent pro-inflammatory innate immune effector functions and, according to the concept of “emergency myelopoiesis”, are suggested to promote increased innate immune surveillance during periods of systemic insult, i.e. trauma and sepsis [48]. According to our data, this may be the case in chronic stress. Our results show that immature granulocytic cells started to expand already two days after first exposure to a dominant male. These immature myeloid cells accumulated preferentially in the red pulp of the spleen and clustered around B cell follicles. Elevated numbers of monocytes and granulocytes in spleen and blood have also been found in other stress models and described as being activated and resistant to glucocorticoid treatment [37;49;50]. Such cells were studied extensively in tumor models and during infections [30–32]. The CSC-induced immature myeloid cells were identified by their morphology as being granulocytic or monocytic in nature and, therefore, can be called PMN-MDSC and MO-MDSC, respectively [1]. The implication of the SNS in the observed CSC-induced cellular changes was demonstrated by chemical sympathectomy, supporting the findings in other models of chronic psychosocial stress [9;10;37]. Signaling of TNF via TNFR2 was also found to be involved in CSC-induced accumulation of suppressor cell types such as Treg cells as well as MDSC, as could be expected from previous data [39–44].

On a functional level, *in vitro* analysis revealed that splenic MO-MDSC produced higher levels of IL-10 and NO_2_^-^ upon LPS stimulation and that bone marrow-derived MO-MDSC showed an enhanced suppression of T cell proliferation when obtained from CSC compared to SHC mice. Also, NO_2_^-^ in the supernatants of those T cell proliferation assays was increased in the CSC compared with the SHC group. Notably, as described above in the introduction, NO_2_^-^ is a well-known mediator of the suppressive effects of MDSC on anti-tumoral T cell responses [24]. The much lower NO_2_^-^ production by spleen- versus bone marrow-derived MO-MDSC during *in vitro* LPS stimulation, independent of CSC exposure, corresponds to their less suppressive *in vitro* effect.

As expected, tumor cells transplanted into CSC mice grew faster in the presence of anti-inflammatory and immune regulatory cell types, i.e. Treg cells and MDSC, and respective tumors were characterized by increased vascularization. MO-MDSC, transient in nature, appear to be “trapped” in the suppressive state during chronic or ongoing inflammation by inflammatory mediators [48] providing a programmed cellular link between increased innate immune surveillance and regulated adaptive immune response. In addition, MDSC-caused inhibition of E-selectin expression prevents tumor-specific T cell traffic into the tumor, thus, contributing a mechanism by which MDSC impair anti-tumor immunity [51].

Both the increased Treg cell frequency and the MDSC-caused suppression of CD4^+^ T cell proliferation were only seen in tumor-bearing CSC mice but not in tumor-free CSC mice 9 days after cessation of CSC. Even enhanced suppressive activity towards CD4^+^ T cells became evident in splenic MO-MDSC from tumor-bearing CSC mice which had not been seen directly after cessation of CSC. This observation might reflect recruitment of CSC induced suppressive bone marrow MO-MDSC into the periphery by tumor implantation where they suppress the sensitive CD4^+^ T cells. The CSC-induced phenotypic changes in the composition of myeloid spleen cells, however, were maintained in both tumor-free as well as in tumor-bearing animals 9 days after termination of CSC/SHC with the increase being even more pronounced in the tumor-bearing mice. This suggests that CSC-induced generation of counter-regulatory cellular mechanisms is transient but sensitive to tumor-induced reinforcement, demonstrating the suspected but complex link between stress and risk for cancer.

These data and previous findings [13] support the idea of promotion of tumor growth by chronic psychosocial stress. Thus, stress-induced “emergency myelopoiesis” might promote increased innate immune activation on the one hand [48], while on the other hand perfectly sets the stage for promoted tumor growth, mediated by inflammation-driven improvement of tumor colonization and vascularization as well as inhibition of adaptive immunity. A pro-tumoral conditioning by CSC might, thus, be caused by the activation of both innate and adaptive immunity by chronic psychosocial stress which is accompanied by the generation of counter-regulatory cellular suppressor mechanisms, consisting of cells such as regulatory T cells and MDSC.

## Supporting Information

S1 Fig Analysis of myeloid splenocytes after CSC. After 19d of CSC/SHC spleen cells were stained for surface expression of CD11b, Gr1, CD11c, MHCII, and Ly6C by flow cytometry and three CD11b^+^ myeloid subpopulations were identified by their different degree of Gr1^+^ staining: CD11b^+^ Gr1^high^ (I), CD11b^+^ Gr1^int^ (II) and CD11b^+^ Gr1^low^ (III) **(A)**. Histograms of population I (upper row), population II (middle row) and population III (lower row) for CD11c, MHCII, and Ly6C staining are shown in in **(B)**.
(TIF)

S2 Fig Gating strategy for flow cytometric analysis of MDSC. Living cells were gated based on FSC/SSC properties and lymphoid and myeloid cell populations were gated according to their expression of B220, CD11b, Ly6G, and Ly6C **(A)**. Representative pictures of isolated CD11b^+^ Ly6G^+^ Ly6C^int^ cells (PMN-MDSC, left), and CD11b^+^Ly6G^-^Ly6C^high^ cells (MO-MDSC, right) are shown (hematoxylin/eosin staining) **(B)**.
(TIF)
