# Supplementary figures and images for: Induction of Suppressor Cells and Increased Tumor Growth following Chronic Psychosocial Stress in Male Mice
Source: PLoS One. 2016 Jul 8;11(7):e0159059. doi: 10.1371/journal.pone.0159059 (PMC4938385; doi:10.1371/journal.pone.0159059)

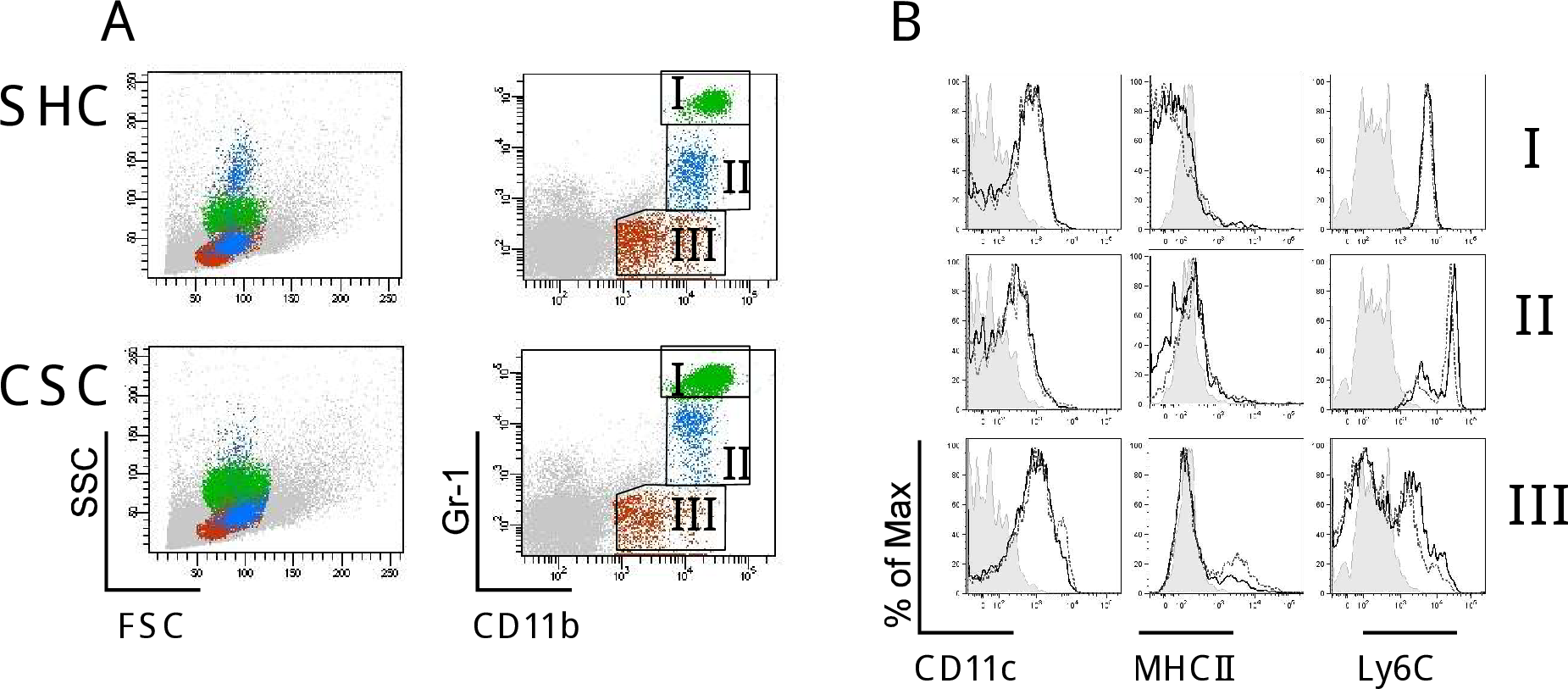

Supplement: S1 Fig — After 19d of CSC/SHC spleen cells were stained for surface expression of CD11b, Gr1, CD11c, MHCII, and Ly6C by flow cytometry and three CD11b+ myeloid subpopulations were identified by their different degree of Gr1+ staining: CD11b+ Gr1high (I), CD11b+ Gr1int (II) and CD11b+ Gr1low (III) (A). Histograms of population I (upper row), population II (middle row) and population III (lower row) for CD11c, MHCII, and Ly6C staining are shown in in (B). (TIF) [file pone.0159059.s001.tif]

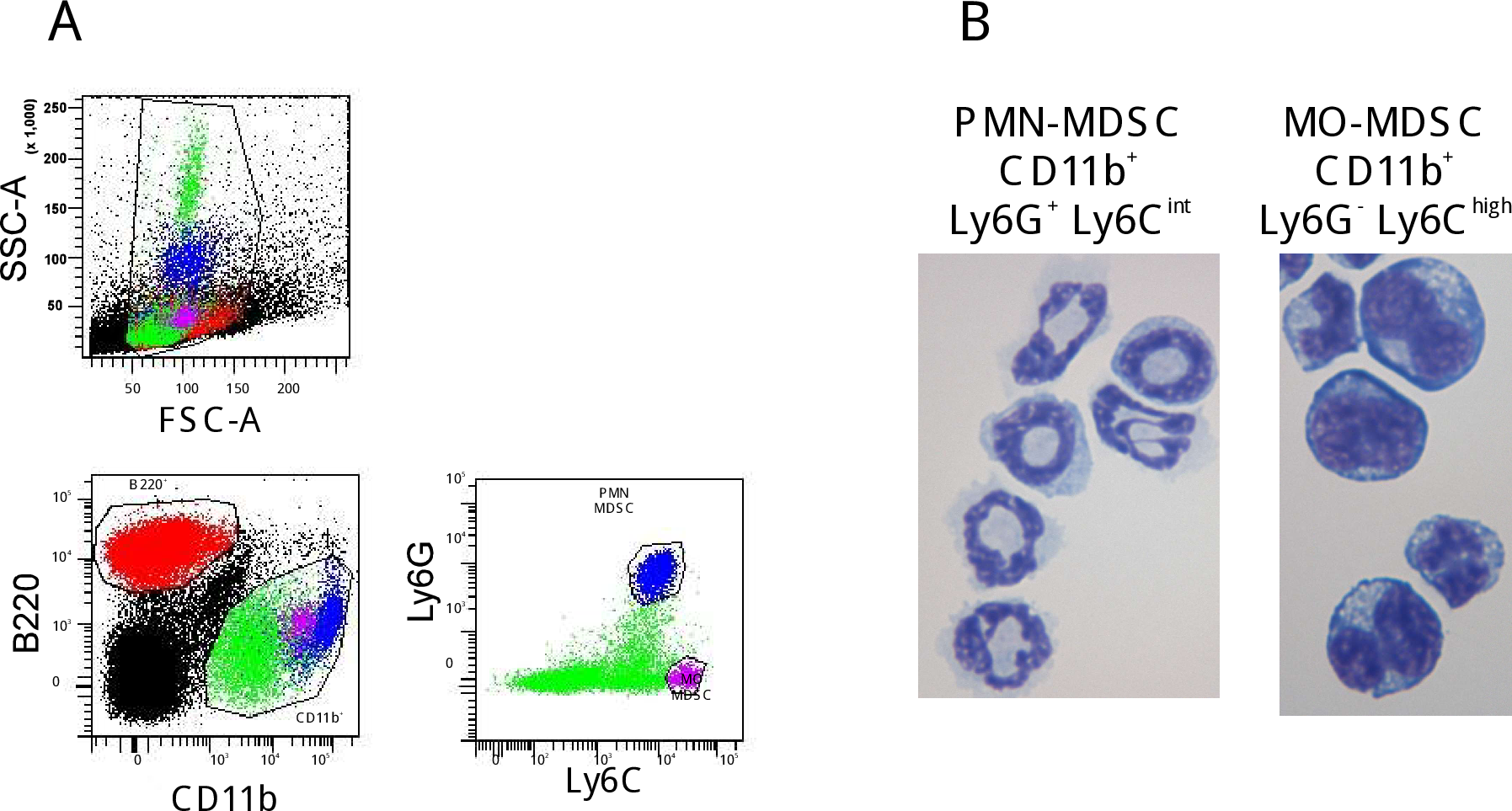

Supplement: S2 Fig — Living cells were gated based on FSC/SSC properties and lymphoid and myeloid cell populations were gated according to their expression of B220, CD11b, Ly6G, and Ly6C (A). Representative pictures of isolated CD11b+ Ly6G+ Ly6Cint cells (PMN-MDSC, left), and CD11b+Ly6G-Ly6Chigh cells (MO-MDSC, right) are shown (hematoxylin/eosin staining) (B). (TIF) [file pone.0159059.s002.tif]
